# The Relationship between Bone Health Parameters, Vitamin D and Iron Status, and Dietary Calcium Intake in Young Males

**DOI:** 10.3390/nu16020215

**Published:** 2024-01-09

**Authors:** Jadwiga Malczewska-Lenczowska, Olga Surała, Dominika Granda, Beata Szczepańska, Adam Czaplicki, Rafał Kubacki

**Affiliations:** 1 Department of Nutrition Physiology, Institute of Sport, National Research Institute, 01-982 Warsaw, Poland; jadwiga.malczewska@insp.pl (J.M.-L.); dominika.granda@insp.pl (D.G.); beata.szczepanska@insp.pl (B.S.); 2 Faculty of Physical Education and Health in Biała Podlaska, Józef Piłsudski University of Physical Education, 00-968 Warsaw, Poland; adam.czaplicki@awf.edu.pl; 3 Faculty of Physical Education & Sport, University School of Physical Education, 51-612 Wroclaw, Poland; rafal.kubacki@awf.wroc.pl

**Keywords:** bone mineral density (BMD), trabecular bone score (TBS), ferritin, free 25-hydroxyvitamin D, adolescent, male

## Abstract

Vitamin D, calcium, and iron are micronutrients crucial for bone health. However, their effect has been studied primarily in the cortical bone, with vitamin D status being assessed mainly from the total 25(OH)D serum fraction. The study aimed to investigate the impact of vitamin D (total and free fraction) and iron status (i.e., serum ferritin or soluble transferrin receptor) and calcium intake (ADOS-Ca questionnaire) on lumbar cortical and trabecular bone. In a cohort of 113 male subjects (76 athletes, 37 non-athletes) aged 15–19, the lumbar spine status (Z-score, bone mineral apparent density (BMAD), and trabecular bone score (TBS)) was determined using dual-energy X-ray absorptiometry (DXA). Relationships between the examined micronutrients and bone health parameters were observed only in athletes. Free 25(OH)D was significantly (*p* < 0.001) correlated with Z-score and BMAD, while total 25(OH)D (*p* < 0.001) and iron status (ferritin, Fe stores; *p* < 0.01) correlated solely with BMAD. Free 25(OH)D and ferritin concentrations were the best determinants of bone status (R^2^ = 0.330; *p* < 0.001) and explained 25% and 7% of the BMAD variance, respectively. No relationships were found between the micronutrients and TBS. The results confirmed the positive influence of vitamin D and iron on cortical, but not trabecular, bone status solely in physically active subjects. In athletes, free 25(OH)D seems to be a superior indicator of bone health to a total 25(OH)D fraction.

## 1. Introduction

Nutrition is one of the paramount modifiable factors affecting bone health. The most biologically relevant micronutrients for bone growth and the accrual of minerals are primarily vitamin D, calcium, and phosphate [1,2], along with some trace elements, e.g., iron [3].

The primary role of vitamin D is to maintain calcium homeostasis in serum, which is crucial for a multitude of metabolic activities, e.g., neuromuscular function [4,5]. Vitamin D enhances the efficiency of intestinal calcium absorption, stimulates the mobilization of calcium stores from bone tissue [6], and conserves them from urine (the reabsorption of Ca in the kidney) [7]. Calcium, in turn, is an inhibitor of the synthesis, and a stimulant of the degradation, of the active form of vitamin D (feedback mechanism) [8,9]. Another element which affects vitamin D and bone metabolism is iron, which participates in collagen synthesis and the conversion of 25(OH)D to its active form [10,11], and it is essential for osteoblast and osteoclast function [12,13]. Studies have demonstrated that iron excess can harm bone mass and mineral content [13,14,15]. However, data on the association between iron deficiency (ID) and bone health are limited and indefinite.

Although the cause-and-effect relationship between vitamin D, especially collectively with calcium and the risk of low-trauma bone fractures, is well supported [9,16,17,18,19], results concerning the relationship between vitamin D status and bone mineral density (BMD) are ambiguous [1,20,21,22,23]. The standard indicator of vitamin D status is the total 25(OH)D concentration in the blood serum, which is almost entirely (~99%) protein-bound, i.e., with vitamin D-binding proteins (VDBPs) and albumins [21,24]. In accordance with the free hormone hypothesis, only an unbound fraction of 25(OH)D may exert biological activity [8,9,25]. Thus, the free fraction of 25(OH)D, rather than total 25(OH)D, is presumably a superior measure of vitamin D status in the context of bone health [21,26]. However, in most research, the bioavailable fraction of vitamin D is calculated (and not measured) based on 25(OH)D total and VDBP. Only recently has it been possible to measure free 25(OH)D directly; hence, it is vital to perform further research concerning the relationship between the directly measured free fraction of vitamin D and the health status of bone tissue.

It should be underlined that the vast majority of research on the effects of nutrients on bones concerns the cortical part of the bones or their metabolism. Meanwhile, only few studies have analyzed the exclusive effect of vitamin D on trabecular bone structure, and the existing results are ambiguous and are mainly derived from animal studies [5,27,28,29].

Another issue is that most research concerns the elderly population. Meanwhile the availability of some nutrients is essential not only for maintaining a healthy skeleton in adults but also for bone growth and development across childhood and adolescence. Data from studies involving young populations are scarce and require greater clarification [4,30]. Therefore, this study aimed to investigate the effects of selected nutritional factors, i.e., vitamin D, iron status, as well as usual Ca intake, on lumbar cortical and trabecular bone in young male subjects.

## 2. Materials and Methods

### 2.1. Subjects

The study was performed during the summer and autumn periods. A total of 121 subjects participated in this study. The inclusion criteria were as follows: (1) male gender; (2) age, 15–19 years old; (3) absence of an injury at the time of the study; (4) those engaged in osteogenic sports (volleyball or ski jumping) and non-athletes (depending on the group). Having considered the exclusion criteria (acute or chronic disease affecting bone health, any long-term immobilization or bone fracture across the 12 months prior to the study, BMI below 15 kg/m^2^ and above 37 kg/m^2^, the presentation of written informed consent signed by subjects or parents and/or guardians of underage participants), the results from 113 subjects were analyzed. The surveyed group included 76 members of Polish national youth teams (28 ski jumpers (ski jumping), 48 volleyball players (volleyball)) and 37 non-athletes who constituted the control group (control). All subjects were students at secondary schools. The athletes represented osteogenic sport disciplines, were at a comparable chronological age, and they did not differ in terms of training experience. The present results are part of a more comprehensive project performed in a group of physically active and non-active male youth. The ethical approval to conduct the study was granted by the Ethics Committee of the Institute of Sport—National Research Institute (KEBN-18-39-JM; 18 May 2018). All participants provided informed consent prior to any experimental procedure. The study conformed to the standards required by the World Medical Association Declaration of Helsinki, 2000.

### 2.2. Procedures

#### 2.2.1. DXA Measurements

The Lunar Prodigy densitometer (GE Healthcare Inc., Madison, WI, USA) and the GE Encore software (version 1.6) were used to obtain the bone densitometry measurement. At the start of each testing day, the DXA scanner was calibrated using a spine phantom. The coefficient of variation (CV%) was 0.6% for the lumbar spine (L1–L4) bone mineral content (BMC), 0.4% for L1–L4 areal bone mineral density (aBMD) and 1.4% for trabecular bone score (TBS).

#### 2.2.2. Lumbar Spine BMAD and TBS Measurements

To classify bone mineralization in pediatric groups, the posterior–anterior (PA) lumbar spine (L1–L4) is preferred for performing BMC (g) and aBMD (g/cm^2^) measurements [31]. According to the Official Pediatric Positions of the International Society for Clinical Densitometry (ISCD, 2019), in children and adolescents, bone mineral apparent density BMAD (not aBMD) is recommended for assessing bone mineralization [32]. Thus, BMAD (g/cm^3^) was calculated as follows: (L1 BMC + L2 BMC + L3 BMC + L4 BMC)/(L1V + L2V + L3V + L4V). For each vertebra, the volume (V) was calculated as the area of the respective vertebra, raised to the 1.5 power [31,32]. Furthermore, when the Z-score values for BMD in adjacent vertebrae differed by more than 1 SD, the outlier result was excluded, and the mean values of BMAD, Z-score and TBS were calculated from 3 vertebrae (ISCD, 2019) [32]. In addition, the clinical age-matched and gender-specific Z-score index for the L1-L4 area was calculated with GE Encore software, using age-appropriate reference values to assess bone mineralization. Due to the lack of reference data, the Z-score was not adjusted for height.

Trabecular bone microarchitecture in L1–L4 was assessed indirectly with TBS iNsight (Osteo) software (Version 3.0.2, Medimaps, Pessac, France), based on measurements of BMD in the lumbar spine.

#### 2.2.3. Blood Assays

Iron and vitamin D status were determined from blood withdrawn from the antecubital vein in the morning (8 a.m. to 9 a.m.) after overnight fasting and a minimum of 12 h after the strenuous physical effort. The following analyses were conducted in serum: soluble transferrin receptor (sTfR) concentration using immunoenzymatic commercial kits (Ramco, Stafford, TX, USA); ferritin concentration using an immunoturbidimetric method (Pentra, Horiba ABX, Montpellier, France); and free and total 25(OH)D (VitD_F and VitD_T, respectively), both using enzyme-linked immunosorbent assays (ELISA kits, DIASource, Louvain-la-Neuve, Belgium). For the sake of higher accuracy, the assays were performed following the manufacturer’s protocol. All serum samples (never frozen or only once frozen (−20 °C)) were investigated in duplicate. The intra-assay variability for those indices was 2.4%, 1.8%, 2.8%, and 2.1%, respectively. The reproducibility of the tests’ performance (between-run precision) for sTfR, ferritin, and free and total 25(OH)D did not exceed 6.0%, 7.5%, 2.7%, and 2.8%, respectively.

In order to exclude subjects with the acute phase reaction in whole-blood samples, the white blood cell count (WBC) (hematology analyzer Sysmex 1000×, Sysmex Corporation, Kobe, Japan), erythrocyte sedimentation rate (ESR) after one hour, and C-reactive protein concentration (CRP) in serum (immunoturbidimetric method, Pentra, Horiba ABX, Montpellier, France) were determined. The CRP reagent used in the study covered a wide range of linearity incorporating both normal (1.0–5.0 mg/L) and inflammatory response ranges (5.0–160.0 mg/L). The intra-assay variability for WBC and CRP was 3.0% and 2.3%, respectively, and between-run precision for CRP did not exceed 4.3%.

All analyses were performed in a laboratory with an implemented quality system at the Institute of Sport—National Research Institute (protocol of accreditation #AB946).

Body iron stores (Fe_S) were evaluated based on ferritin and sTfR concentrations, using the algorithm [33] developed exclusively for sTfR determined by the Ramco method.

#### 2.2.4. Intake of Dietary Calcium

The calcium intake from the diet was determined using a validated semi-quantitative food frequency questionnaire (Dairy Products Frequency Questionnaire—ADOS_Ca) [34]. The questionnaire was completed after training in the interviewer’s presence and assistance. Briefly, the data on the frequency of intake of 11 groups of dairy foods (across the preceding six months) were collected. Respondents were also surveyed about typically eaten servings. The calcium intake from each dairy product was calculated. The total daily calcium intake (mg/day) from dairy products was assessed by summing the calcium intakes from all groups of dairy products according to the formula developed in the validation study [34].

#### 2.2.5. Body Composition

All measurements were performed in the morning (8:00–9:00 a.m.) after a roughly 12 h overnight fast. Body composition was estimated using a bioelectrical impedance analysis method (BIA; Tanita BC-420MA, Tokyo, Japan). The participants were measured wearing only underwear, with all metal objects removed and being bladder-voided. The following body components were calculated: fat mass (FM), fat-free mass (FFM), and muscle mass (MM). Body height was measured using a stadiometer (Seca 285, Seca, Hamburg, Germany). BMI was calculated as weight (kg) divided by height squared (m^2^).

#### 2.2.6. Bone Fracture

Data on the frequency of bone fractures in the past (at least 12 months previously) were collected using the authors’ questionnaire.

#### 2.2.7. Statistical Analyses

The normality of the distributions and homogeneity of the variances of the empirical data in the studied groups were checked using Shapiro–Wilk and Levene tests.

Depending on the results of these tests, a series of one-way analyses of variance (ANOVA) or non-parametric tests (Kruskal–Wallis or Mann–Whitney) were performed to detect differences between mean/median values in each of the three groups (ski jumping, volleyball and control). The significance of these differences between groups was tested using the Tukey or Dunn test. In order to establish analytical relationships between variables in the studied groups, correlations between individual quantitative variables were examined first. Some pairs of variables were not bivariate normal, and because of the possible non-linear nature of the relationship between variables, the Spearman rank correlation coefficient was used.

Due to the same direction and magnitude of the relationship between nutritional factors and mineral bone density in ski jumpers and volleyball players, the results of the correlation analysis are presented in the combined group (sport group = ski jumping + volleyball).

**Figure 1:**
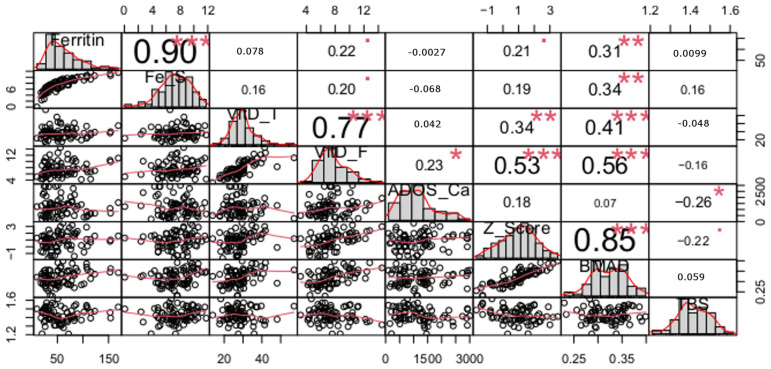
Spearman correlation coefficients for the sport group (Significance level of correlation coefficients: * *p* < 0.05, ** *p* < 0.01, *** *p* < 0.001; Fe_S (Fe stores), VitD_T (total 25(OH)D), VitD_F (free 25(OH)D), ADOS_CA (dietary calcium intake).).

**Figure 2:**
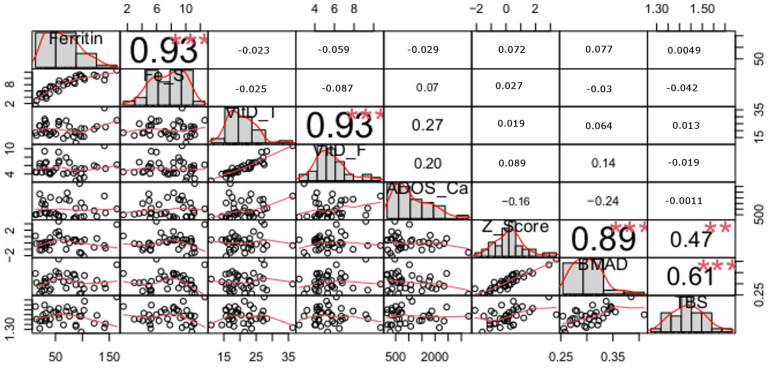
Spearman correlation coefficients for the Control group (Significance level of correlation coefficients: ** *p* < 0.01, *** *p* < 0.001; Fe_S (Fe stores), VitD_T (total 25(OH)D), VitD_F (free 25(OH)D), ADOS_CA (dietary calcium intake).).

The basis for combining volleyball players and ski jumpers into one sport group was also the osteogenic nature of physical efforts in both disciplines as well as the similar age and training experience of the subjects. Subsequently, the form of the analytical relationships between the dietary variables (ferritin, Fe_S (body iron stores), VitD_T (total 25(OH)D), VitD_F (free 25(OH)D), and ADOS_CA (dietary calcium intake)), the variables of bone mineralization, i.e., BMAD and Z_Score (Z-score) (see Figure 1 and Figure 2), and the trabecular bone score (TBS) were determined using multiple regression. The calculations used a bidirectional stepwise regression algorithm allowing both the addition and deletion of variables from the model in successive iterations. The significance of the relationship between the dichotomous variable “Fractures” and the other variables was verified by means of logistic regression. Statistical calculations were performed in the R environment (R Foundation for Statistical Computing, Vienna, Austria). Statistical significance in all analyses was set at *p* < 0.05. For this level of significance, the power of ANOVA and non-parametric tests was calculated. In the second case, the MultNonParam package was used in the computations.

## 3. Results

The general characteristics of the subjects are presented in Table 1. Both groups of athletes did not differ in age, training experience, BMI (kg/m^2^), or fat mass (kg), but they differed significantly in body mass (kg), stature (m) and fat mass status (%). The control group, in turn, was characterized by higher BMI and greater fatness (in percentage and kilograms) and was older than both sports groups.

The mean values of the analyzed quantitative variables and number of fractures in the sport group (ski jumping and volleyball collectively) vs. the control group are presented in Table 2, while Table 3 presents the results of the univariate analysis of variance and the results of the non-parametric tests for these variables.

Comparing the two groups, i.e., sport vs. control, large effect sizes were evident for total 25(OH)D and medium effect sizes were evident for free 25(OH)D, and a small effect was observed for Z-score but not for the rest of the indices (ferritin, Fe stores, calcium intake, BMAD, and TBS).

Among the subjects, 66% of athletes and 98% of controls demonstrated deficiency (<20 ng/mL) or insufficiency (20–30 ng/mL) of vitamin D (25(OH)D) [37]. In the case of iron status, a ferritin concentration < 30 ng/mL was detected in 22% of athletes and 16% of controls. The percentage of subjects with usual dietary calcium intake below the age-adjusted Estimated Average Requirement for the Polish population [38] was 62% in the control group and 49% in the sport group.

The results of the correlation analysis between quantitative variables for the sport and control groups are presented in Figure 1 and Figure 2, respectively.

High correlations between pairs of variables related to iron status (ferritin and Fe stores), bone mineralization indices (BMAD and Z-score) and two indices of vitamin D status (total 25(OH)D and free 25(OH)D) in both the sport and control groups were apparent. However, the differences between the coefficients of correlation for the total and free 25(OH)D in the sport and control groups were statistically significantly different (*p* < 0.01). This correlation was significantly stronger in the controls than in the athletes.

High correlations between nutritional factors and bone indicators were observed only in the sport group and concerned only indices of bone mineralization (not with trabecular structure). Thus, in athletes, free 25(OH)D are significantly correlated with both Z-score and BMAD. In the case of total 25(OH)D and two indices of iron status (ferritin and Fe Stores), those correlations were weaker although still significant with BMAD (not with Z-score). Daily calcium intake from dairy products (ADOS_Ca) did not correlate with either indices of bone mineralization or trabecular structure.

In the control group, TBS correlated with both Z-score and BMAD, which was higher in the latter case. In the sport group, however, such a correlation was not observed.

The multiple regression results are presented in Table 4. The results were found to be most statistically significant in the sport group for the model BMAD~ferritin + free 25(OH)D. Both variables explained a total of 32% of BMAD variation. The free fraction of vitamin D made the most significant unique contribution to the regression equation (explaining 25% of BMAD variation), while ferritin explained the remaining 7% of BMAD variability.

In the control group, there were no statistically significant relationships between nutritional factors and the indices of bone mineralization.

None of the logistic regression models tested proved statistically significant for the dichotomous variable “Fractures” as a function of the quantitative variable or a combination of these variables. The “Fractures” variable was also found to be independent of the categorical group variable.

## 4. Discussion

This study aimed to investigate the impact of selected nutritional factors, i.e., vitamin D and iron status as well as usual calcium intake, on bone mineralization and its trabecular structure in young (adolescent and early adulthood) male subjects.

The presented results confirmed the influence of vitamin D and iron status on bone mineralization, although only in physically active subjects. In this group, both nutritional factors explained 32% variability in bone mineralization expressed by BMAD. The results, however, did not demonstrate any influence of the studied nutritional factors on the TBS.

### 4.1. Nutrients and Bone Mineral Density

Our results in a group of physically active males showed a positive relationship between BMAD and Z-score and the level of vitamin D in serum measured as total and free fraction. It is worth noting, however, that the relationship between those bone indices and the concentration of 25-hydroxy vitamin D was stronger in the case of the free fraction than the commonly used total fraction (*p* = 0.53; *p* < 0.001 and 0.56; *p* < 0.001 vs. 0.34; *p* < 0.01 and 0.41; *p* < 0.001, respectively).

Multiple regression analysis showed that free 25(OH)D definitely has the largest unique contribution towards the variability in bone density in the sport group. This variable explained 25% of the total 32% of BMAD variation in the lumbar spine. For comparison, in studies of Finnish children and adolescents, it was found that 25(OH)D concentration explained only 5.6% of the variance in lumbar spine BMD, but the vitamin D status was assessed using the total fraction of 25(OH)D, and lumbar bone density was not adjusted for height (it was expressed with BMD) [39]. Our results suggest that in physically active subjects, free 25(OH)D may be a more specific indicator of vitamin D status in the evaluation of bone density than the commonly used total 25(OH)D. This observation is consistent with the results of other authors [21,40]. Johnsen et al. (2014) concluded that the free and bio-available forms of 25(OH)D may be a more informative measure of vitamin D status than total 25(OH)D concerning bone tissue [40] Also, Allison et al. (2018) pointed out that the choice of total 25(OH)D is not fit for practice when examining the relationship between bone health and vitamin D concentrations in athletes [21], while Lieben and Carmeliet (2013) emphasized that the free 25(OH)D fraction has a more significant biological effect on target cells, regulating calcium absorption in the intestines, calcium resorption in bones and calcium resorption in the kidneys [41].

It is intriguing and, at the same time, challenging to explain the lack of relationship between vitamin D metabolites (free and total) and bone mineralization indicators in the group of non-training subjects (control group), especially since the percentage of controls with insufficient (20–30 ng/mL) and/or deficient (<20 ng/mL) total 25(OH)D concentration was much higher than in athletes (98% vs. 66%; 49% vs. 8%). Since healthy bones are the result of a long formation process and the nutritional status of vitamin D reflects the current state, it cannot be ruled out that observed differences in the relationship between vitamin D status and bone mineralization in both study groups could be the result of different levels of the body’s supply of this vitamin in the past [42]. The size of the control group did not affect the observed differences in the relationship between bones and vitamin D status because additional analyses (presented in Supplementary Materials) comparing the control group to ski jumpers and separately to volleyball players gave the same result.

What is noteworthy in the obtained results is the significantly different relationship between the free and total 25(OH)D fraction in the studied sport and control groups. The free 25(OH)D fraction constitutes 0.1–2% of the total hydroxy vitamin D in the human body. The rest circulates in the blood bound to proteins, mainly VDBP and less by albumins [43], so theoretically, the relationship between both metabolites should be similar in all subjects. Meanwhile, the correlation between free 25(OH)D and total 25(OH)D in the athletes was lower (coefficient of correlation: sport group—0.77 vs. control group—0.93). Therefore, physical exercise presumably affects the total 25(OH)D fraction by influencing the concentration of VDBP. It is known that changes in the VDBP concentration may affect the total 25(OH)D concentration [44]. VDBP, as a negative active-phase protein, may be lowered during inflammation [45] and, consequently, may have an influence on the reduction in the total 25(OH)D concentration. The control of acute-phase indicators in the current study allowed us to eliminate subjects with visible symptoms of inflammation; however, in the athletes, heavy physical exercise itself may cause inflammation at the level of muscle cells [46]. Nevertheless, the available data concerning the influence of physical exercise on total 25(OH)D are equivocal, as some studies have found an increase in this metabolite under the influence of acute bouts of endurance exercise [47]. Therefore, in competitive athletes, the influence of physical exercise on the total 25(OH)D concentration cannot be ruled out. Regardless of the direction, these changes could have reduced the relationship between total and free 25(OH)D fractions in the sport group.

In addition to vitamin D, bone homeostasis requires optimal iron levels. It is known that iron overload undoubtedly increases osteoclast differentiation and activity as well as inhibiting osteoblast differentiation and function, which can manifest in lower BMD and in altered microarchitecture and biomechanics [12]. Iron excess was not detected in our study; however, 22% of the athletes and 15% of the controls were iron-deficient. Both ferritin and iron stores simultaneously correlated with BMAD, but not with the Z-score, exclusively in the sport group. Studies which demonstrated a direct effect of iron deficiency on unbalanced bone turnover were conducted mainly on animal models [48,49]. The results of one study involving humans [50] suggested that iron deficiency may be linked to higher bone loss, which was based on a negative correlation of ferritin with the biomarker of bone resorption in women. Another human-model study analyzing the relationship between iron status and bone mineralization showed a negative correlation between ferritin and mineral bone density in older women [51]. Also, the study by Peng et al. (2022) conducted in a large female population indicated a negative association between serum ferritin and lumbar spine BMD in females over 45 [52]. The mentioned outcomes are in accordance with our results, at least in the sport group. It should be emphasized, however, that all human studies cited above concerned a different population, i.e., adult women.

The lack of correlation between indices of iron status and Z-score in our study may be due to the lack of adjustment of this indicator to height (Z-score is normalized only for healthy male peers). BMAD on the other hand is a parameter adjusted for height, which partially reduces the confounding influence of shorter stature on bone density [31,53]. Multiple regression analysis showed that besides the abovementioned free 25(OH)D, ferritin is an indicator which has a significant impact on the lumbar spine density (as it explained the remaining 7% of BMAD variability). Those results suggest that ferritin could be more precise in physically active subjects than the calculated iron stores in assessing bone mineralization, despite the level of correlation between both iron indices and BMAD being comparable (0.31, *p* < 0.01 and 0.34, *p* < 0.01).

The results of the current study did not show that the usual calcium intake had an impact on BMAD in both groups, which is consistent with the results of others indicating that (1) dietary calcium intake itself is a less important (although still significant) predictor of total body bone mass [54]; (2) seasonal fluctuations in calcium absorption in the intestines do not affect bone turnover [42]; (3) additional intake of dairy products has a small effect on bone mineral mass in adolescents [55].

Typical calcium intake does not always reflect normal calcium status, primarily because of the varying degree of its absorption in the gastrointestinal tract, which depends mainly on vitamin D status. Although severe vitamin D deficiencies were not observed among the participants, the frequency of decreased values of total 25(OH)D was high in both groups, which could have influenced calcium absorption. Data indicate that even moderate, prolonged vitamin D deficiency may impair intestinal calcium absorption, causing in sequence hypocalcemia, an increase in serum parathyroid hormone (PTH) concentration (secondary hyperparathyroidism), accelerated bone turnover and ultimately increased bone loss and increased risk of fractures [6,8,9,56,57,58].

On the other hand, in adolescents, an additional factor exists—Nicolaysen’s endogenous factor, which facilitates calcium absorption and allows higher calcium requirements to be met [59]. Furthermore, during puberty, low calcium intake may be compensated by high calcium retention efficiency in the body [54], which was confirmed by the results of other studies indicating a low influence of calcium intake on its urinary excretion in adolescents [59]. There is also the concept that calcium is a threshold nutrient—when a plateau is reached, a higher amount of absorbed calcium does not cause further gain in calcium retention, irrespectively of vitamin D level [60].

Therefore, to assess the effect of calcium on bone tissue, it is worth evaluating the calcium balance, considering its intake, absorption, retention, and excretion. The lack of correlation between the amount of calcium consumed in the diet and the state of mineralization of bone tissue in our study may be a confirmation of this.

### 4.2. Nutrients and Trabecular Bone Structure

Our research found no relationship between the trabecular structure of bones and the analyzed nutritional factors. So far, little research has been conducted on the effect of dietary factors on trabecular bone. Most studies have been performed on animals; their results are ambiguous and concern only the effect of vitamin D. Earlier results on rats indicated that alfacalcidol, an analogue of vitamin D, improves both trabecular and cortical sites of bone [28]. The latest studies on rats indicated that vitamin D alone, without the support of medications for osteoporosis, is not effective in improving trabecular bone [5]. The latest human study showed a supportive effect of vitamin D on the trabecular structure in young adults, indicating an association between low vitamin D and poorer bone microarchitecture and bone strength. However, it is worth noting that the measurements were performed in a different bone area than in our study, i.e., the distal radius and tibia (not in the lumbar spine) [29]. Notably, the relationship between the TBS and bone mineralization indexes differed between the study groups. TBS correlated with both Z-score and BMAD in the control group, while no such correlation existed in the sport group. At the same time, the mean values of TBS and BMAD were similar to those in the control group, implying an influence of other factors on bone-building processes in athletes. The studied athletes represented disciplines where the share of antigravity efforts (jumps, landings) on bone tissue is very high. Mechanical forces are a vital regulator of osteoblast and osteoclast activity and support maintaining the integrity of the skeletal system. The task of trabecular bone is to transfer the load from joints to the compact bone of the cortex of long bones, and in the vertebral bodies, it represents the main load-bearing structure [61]. Osteocytes, which are sensitive to mechanical loads, are considered to control the process of adaptive remodeling of bone by regulating osteoblast and osteoclast function. Although results obtained in animals should not be extrapolated directly to humans, the data indicate that, for example, a jumping routine causes changes in the trabecular structure of the femur consisting of a thickening of the trabeculae, and not an increase in their number [62].

Even though both BMAD and Z-score correlated with the TBS in the controls, the correlation coefficients differed. The significantly higher correlation with BMAD (*p* < 0.001) than with Z-score (*p* < 0.01) may result from the correction of aBMD for the height of all subjects, after which the mean values of BMAD became similar in athletes and controls. In contrast, the mean values of Z-scores differed between the groups. These results confirm the official position of the ISCD that, in adolescents with different body heights, the BMAD index should be considered when assessing bone mineralization [31].

### 4.3. Frequency of Fractures

Data from the research conducted so far indicate that adolescent fractures are associated with reduced BMC and BMD and may be a marker of skeletal fragility [31,63]. In our study, the “Fractures” factor was comparable in examined groups and independent of mineralization and TBS in the lumbar spine. Moreover, the similar incidence of fractures in both study groups suggests that it may be a factor independent of the level of physical activity and associated overloads promoting progressive micro-injuries [64]. Considering the character of both disciplines, it cannot be completely ruled out that a large share of osteogenic efforts, especially antigravity ones, such as jumping up and down may translate to improved bone status [65]. Also, none of the studied nutritional factors impacted the incidence of fractures in the past in both groups, although in the sport group, vitamin D and iron status affected bone mineralization.

### 4.4. Limitations and Strengths

The presented study has some strengths but also some limitations. The strengths are as follows: (1) the simultaneous and direct measurement of free and total fractions of 25(OH)D in serum; (2) the measurement of the mineralization and trabecular structure of bone tissue; (3) a proper (recommended by the ISCD in adolescents) assessment of bone mineralization based on BMAD; (4) the assessment of iron status based on two indices, i.e., ferritin and calculated total iron stores; (5) usual calcium intake was assessed with a validated tool; (6) the study concerned only healthy athletes without any symptoms of the acute phase reaction; (7) a simultaneous analysis of the three nutritional factors most important for bone and at the same time interdependent; (8) the study involved a group of adolescent males with diverse physical loads, i.e., professional athletes and non-training subjects (control group).

The limitations include the relatively small sample, which limits the generalizability of the reported findings. However, it is difficult to find a representative number of subjects from one sports discipline with similar training experience and sports level who are concurrently of the same age. This was one of the reasons to combine representatives of both sports disciplines into one sport group. The Materials and Methods section further explains why volleyball players and ski jumpers were combined into one group.

Bone metabolism is a process requiring the constant presence of nutrients, but measurements of serum vitamin D and iron nutritional status indicators offer a snapshot of only the current status. An additional limitation is that only males were studied. Female athletes may present more advanced deficiencies, especially in terms of iron, which could influence bone status to a greater extent. Apart from that, the cross-sectional nature of this work made it impossible to assess causal relationships between nutritional factors and bone parameters and thus draw more unequivocal conclusions.

## 5. Conclusions

The results confirmed the positive influence of proper/normal vitamin D and iron status on cortical, but not trabecular, bone status solely in physically active subjects. In terms of assessing bone mineralization, the free 25(OH)D fraction seems to be a better indicator than total 25(OH)D, and ferritin seems to be more useful in this regard than calculated body iron stores.

An additional conclusion from the current research (beyond nutritional factors) points to a need to assess bone mineralization in adolescents based on adjusted-for-height BMAD (not raw aBMD data). The results also indicate that, in athletes, trabecular bone assessed based on the TBS parameter is independent of bone mineralization, although an additional impact of osteogenic physical exercises on this cannot be excluded.

## Figures and Tables

**Table 1:** General characteristics of the study sample (mean ± SD).

Variables	Control *n* = 37	Ski Jumping *n* = 28	Volleyball *n* = 48	*p* Value
Age (years)	18.1 ± 1.4 *	16.7 ± 1.2	17.3 ± 10.8	~0.000
Body mass (kg)	75.6 ± 12.0 ^^^	57.7 ± 7.0 ^^^	84.2 ± 9.2 ^^^	~0.000
Height (m)	1.81 ± 0.08 ^^^	1.74 ± 0.06 ^^^	1.96 ± 0.07 ^^^	~0.000
BMI (kg/m^2^)	23.8 ± 3.3 *	20.0 ± 1.4	22.0 ± 2.0	~0.000 ^KW^
Fat mass (%)	15.2 ± 5.6 ^^^	7.18 ± 2.40 ^^^	10.8 ± 2.7 ^^^	~0.000 ^KW^
(kg)	12.4 ± 6.0 *	4.26 ± 1.79	9.2 ± 2.8	~0.000 ^KW^
Fat-free mass (kg)	65.2 ± 7.1 ^^^	53.4 ± 5.6 ^^^	75.1 ± 7.6 ^^^	~0.000
Muscle mass (kg)	62.0 ± 6.8 ^^^	50.7 ± 5.4 ^^^	71.4 ± 7.3 ^^^	~0.000
Training experience (years)	-	7.37 ± 2.18	6.48 ± 2.86	0.09 ^MW^

*—significantly different from the other groups; ^^^—all groups differed from each other; BMI—body mass index, ^KW^—Kruskal–Wallis test, ^MW^—Mann–Whitney test.

**Table 2:** Mean values ± SD of the variables under study.

	Group	
Variable	Control	Sport (Ski Jumping + Volleyball)
	*n* = 37	*n* = 76
Ferritin (ng/mL)	67.2 ± 39.9	58.3 ± 34.0
Fe stores (g)	7.72 ± 2.3	7.5 ± 2.2
Total 25(OH)D (ng/mL)	21.0 ± 5.0	28.5 ± 7.1
Free 25(OH)D (ng/mL)	5.50 ± 1.8	7.6 ± 2.3
Calcium intake (mg/day)	1039 ± 767	1207 ± 743
Z-score	0.11 ± 1.14	0.89 ± 1.16
BMAD (g/cm^3^)	0.31 ± 0.03	0.32 ± 0.04
TBS	1.433 ± 0.08	1.426 ± 0.08
Fractures (%)	22	34

Fe stores—body iron stores; total 25(OH)D—total fraction of 25(OH)D; free 25(OH)D—free fraction of 25(OH)D; BMAD—lumbar spine bone mineral apparent density; TBS—trabecular bone score; fractures—percentage of subjects with at least 1 fracture in the past.

**Table 3:** Results of one-way analysis of variance and non-parametric tests for groups studied.

		Control vs. Sport Group (Ski Jumping + Volleyball)
Variable	*p*	ES ^#^
Ferritin	NS	
Fe stores	NS	
Total 25(OH)D	*	ε^2^ = 0.285; CI (0.161; 0.428)
Free 25(OH)D	*	ε^2^ = 0.206; CI (0.078; 0.348)
Calcium intake	NS	
Z-score	*	η^2^ = 0.09; CI (0.021; 0.161)
BMAD	NS	
TBS	NS	
Fractures	NS	

Fe stores—body iron stores; total 25(OH)D—total fraction of 25(OH)D; free 25(OH)D—free fraction of 25(OH)D; BMAD—lumbar spine bone mineral apparent density; TBS—trabecular bone score. CI—confidence interval. Significant differences: *—*p* < 0.001; NS—not significant. ^#^—epsilon squared (ε^2^) effect sizes (ESs) were taken as small for 0.01 ≤ ε^2^ <0.08, medium for 0.08 ≤ ε^2^ < 0.26, and large for ε^2^ ≥ 0.26 and those for eta squared (η^2^) were taken as small for 0.01 ≤ η^2^ <0.06, medium for 0.06 ≤ η^2^ < 0.14, and large for η^2^ ≥ 0.14 [35,36]. The power of ANOVA was 0.91.

**Table 4:** Results of multiple regression analysis and a unique contribution of predictor variables for BMAD variation in the sport group.

Variable	Nonstandard Coefficients		Standard Coefficients			Unique Contribution
	Beta	SE	Beta	t	*p*	U
Intercept	0.246	0.013		19.38	<0.001	
Ferritin	0.0002	0.0001	0.227	2.32	<0.05	0.07
Free 25(OH)D	0.008	0.0015	0.486	4.97	<0.001	0.25

F(2,73) = 18.22, *p* < 0.001, R^2^ = 0.33.

## Data Availability

Data are contained within the article and Supplementary Materials.

## Supplementary Materials

The following supporting information can be downloaded at: https://www.mdpi.com/article/10.3390/nu16020215/s1, Table S1: Mean values ± SD of the variables under study.; Table S2: Results of one-way analysis of variance and non-parametric tests for groups studied.
