# Supplementary material for: The Relationship between Bone Health Parameters, Vitamin D and Iron Status, and Dietary Calcium Intake in Young Males
Source: Nutrients. 2024 Jan 9;16(2):215. doi: 10.3390/nu16020215 (PMC10818825; doi:10.3390/nu16020215)
Supplement: Supplementary file 1 [file nutrients-16-00215-s001.zip › nutrients-2768825-supplementary.pdf]

Table S1. Mean values ± SD of the variables under study.

| **Variable** | Control<br>n=37 | Ski jumping<br>n=28 | Volleyball<br>n=48 |
|---|---|---|---|
| Ferritin (ng/ml)) | 67.2±39.9 | 59.4±40.9 | 57.7±29.7 |
| Fe stores (g) | 7.72±2.3 | 7.6±2.2 | 7.4±2.2 |
| Total 25(OH)D (ng/ml) | 21.0±5.0 | 28.9±8.1 | 28.3±6.6 |
| Free 25(OH)D (ng/ml) | 5.50±1.8 | 6.7±1.9 | 8.1±2.4 |
| Calcium intake (mg/day) | 1039±767 | 858±420 | 1410±816 |
| Z-score | 0.11±1.14 | -0.03±0.97 | 1.43±0.90 |
| BMAD (g/cm$^3$) | 0.31±0.03 | 0.31±0.04 | 0.33±0.03 |
| TBS | 1.433±0.08 | 1.479±0.06 | 1.396±0.08 |
| Fractures (number) | 21 | 13 | 20 |

Fe stores – body iron stores; Total 25(OH)D – total fraction of 25(OH)D, Free 25(OH)D – free fraction of 25(OH)D; BMAD – lumbar spine bone mineral apparent density; TBS – trabecular bone score.

Table S2. Results of one-way analysis of variance and non-parametric tests for groups studied

| | **Control vs. Ski jumping vs. Volleyball** | |
|---|---|---|
| **Variable** | *p* | ES |
| Ferritin | NS | |
| Fe stores | NS | |
| Total 25(OH)D | *** | $\varepsilon^2$= **0.285**; CI<0.161;0.434><br>(C vs. S_J)$^{***}$; (C vs. V)$^{***}$ |
| Free 25(OH)D | *** | $\varepsilon^2$= **0.257**; CI<0.133;0.413><br>(C vs. S_J)$^{*}$; (C vs. V)$^{***}$; (S_J vs. V)$^{*}$ |
| Calcium intake | *** | $\varepsilon^2$= 0.087; CI<0.019;0.222><br>(C vs. V)$^{*}$; (S_J vs. V)$^{*}$ |
| Z-score | *** | $\eta^2$= **0.323**; CI<0.219;0.405><br>(C vs. V)$^{***}$; (S_J vs. V)$^{***}$ |
| BMAD | * | $\eta^2$= 0.063; CI<0.009;0.123><br>(C vs. V)$^{*}$ |
| TBS | *** | $\eta^2$= **0.161**; CI<0.063;0.249><br>(C vs. S_J)$^{*}$; (S_J vs. V)$^{***}$ |
| Fractures | NS | |

Fe stores – body iron stores; Total VitD – total fraction of 25(OH)D, Free 25(OH)D – free fraction of 25(OH)D; BMAD – lumbar spine bone mineral apparent density; TBS – trabecular bone score.

C stands for Control, S_J for Ski_jumping, V for Volleyball, CI – confidence interval. Significant differences: $^{*}$ $p < 0.05$; $^{***}$ $p < 0.001$; NS - not significant.

$^{\#}$Effect sizes (ES) epsilon squared ($\varepsilon^2$) were taken as small for $0.01 \leq \varepsilon^2 < 0.08$, medium $0.08 \leq \varepsilon^2 < 0.26$, large $\varepsilon^2 \geq 0.26$ and for eta squared ($\eta^2$) as small $0.01 \leq \eta^2 < 0.06$, medium $0.06 \leq \eta^2 < 0.14$ and large $\eta^2 \geq 0.14$ [35,36].
